# Diagnostic value of the corrected QT difference between leads V1 and V6 in patients with acute pulmonary thromboembolism

**DOI:** 10.1097/MD.0000000000008430

**Published:** 2017-10-27

**Authors:** Seong Jun Park, Chang Hee Kwon, Byeong Joo Bae, Bum Sung Kim, Sung Hea Kim, Hyun-Joong Kim, Hweung Kon Hwang, Sang-Man Chung

**Affiliations:** The Department of Internal Medicine, Konkuk University Medical Center, Konkuk University School of Medicine, Seoul, Korea.

**Keywords:** corrected QT difference, diagnosis, electrocardiography, pulmonary embolism

## Abstract

In acute pulmonary thromboembolism (PTE), right ventricular pressure overload impairs right-sided cardiac conduction and repolarization. We hypothesized that if heterogeneity of repolarization between right and left ventricles occurs in acute PTE, there would be the difference of repolarization between them. Therefore, we aimed to evaluate the diagnostic value of corrected QT interval (QTc) difference between leads V1 and V6 (V1 − V6) in patients with acute PTE.

A total of 89 patients with suspected acute PTE who underwent computed tomographic angiography (CTA) were enrolled from January to December 2015. PTE was identified by CTA. We compared electrocardiographic (ECG) parameters, especially QTc difference (V1 − V6) between patients with PTE and those without PTE.

Acute PTE was finally diagnosed in 45 patients. Clinical situations including the chief complaint were not different between PTE and non-PTE groups. S1Q3T3, a traditional ECG marker, had no diagnostic value for acute PTE. Patients with PTE had a significantly longer mean QTc in V1 (454.6 ± 44.3 vs 417.5 ± 31.3 ms, *P* < .001) and larger QTc difference (V1 − V6) (34.8 ± 30.5 vs –12.5 ± 16.6 ms, *P* < .001) than non-PTE controls. QTc difference (V1 − V6) was negative in all patients without PTE. PTE patients had a higher prevalence of T wave inversion in leads III (51.1% vs 29.5%, *P* = .038) and V1 (82.2% vs 38.6%, *P* < .001). A QTc difference (V1 − V6) of ≥20 ms identified PTE with 82.2% sensitivity, 100.0% specificity, and 100.0% positive predictive value.

QTc difference (V1 − V6) had an excellent diagnostic value for differentiating patients with and without acute PTE.

## 1 Introduction

Acute pulmonary thromboembolism (PTE) is a common and sometimes fatal disease.^[1,2]^ It has various clinical presentations, such as chest pain/discomfort, dyspnea/dyspnea on exertion, or syncope.^[3–5]^ Thus, it is challenging to diagnose acute PTE based on the clinical situation. Although computed tomographic angiography (CTA) has been preferred as a definitive diagnostic test for acute PTE,^[6]^ 12-lead electrocardiography (ECG) is a simple, prompt, and widely used technique for initial examination for suspected PTE in the acute clinical setting.^[7–9]^

Several ECG abnormalities, such as the S1Q3T3 pattern, negative T waves in the precordial leads, right bundle branch block, and atrial arrhythmias are known to be of diagnostic and prognostic value for PTE.^[8,10–12]^ These ECG findings are associated with right ventricular (RV) strain followed by rapid RV pressure overload. We hypothesized that if the RV strain impairs right-sided cardiac conduction and repolarization,^[13]^ there would be heterogeneity of repolarization between the right and left ventricles in acute PTE. Therefore, we aimed to evaluate the diagnostic value of corrected QT interval (QTc) difference between leads V1 and V6 (V1−V6) in patients with suspected acute PTE.

## 2 Methods

### 2.1 Study patients

The retrospective study involved 101 consecutive patients with suspected acute PTE who underwent CTA at the Konkuk University Medical Center (Seoul, Korea) from January to December 2015. The decision to perform CTA to exclude or confirm PTE was taken by the attending physician on the basis of the patient's clinical situation. Twelve patients were excluded from the study (7 with no available baseline ECG and 5 with unmeasurable ECG). Finally, 89 patients were included in this analysis. PTE was diagnosed if there was thrombus in the pulmonary arteries on CTA. Diagnosis of PTE was decided by radiologist. The principal criteria for categorizing massive PTE were arterial hypotension (systolic blood pressure <90 mm Hg) and cardiogenic shock requiring inotropic or ventilator.^[14]^

This study was approved by the Institutional Review Board of the Konkuk University Medical Center (protocol no. KUH1010821). The requirement for informed consent was waived as de-identified information was retrieved retrospectively.

### 2.2 Electrocardiography

**Figure 1:**
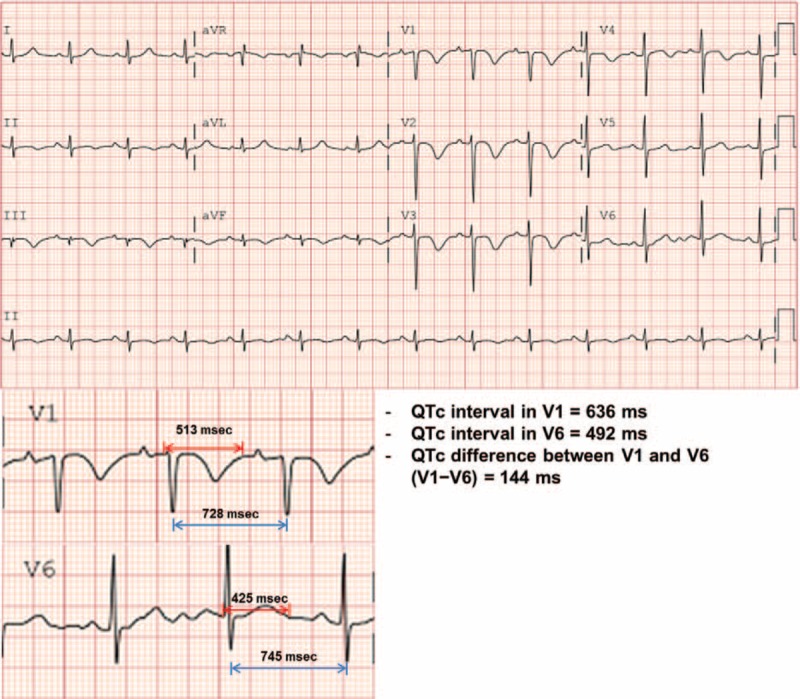
Measurements of QTc intervals in V1 and V6, and QTc difference between V1 and V6 (V1−V6).

Baseline 12-lead ECGs were recorded on admission at a gain of 10 mm/mV and a paper speed of 25 mm/s. T wave inversion was considered present if the depth was at least 1.0 mm. Q or S waves were considered present if the depth was at least 1.5 mm. P pulmonale was defined as P waves with an amplitude of ≥2.5 mm in the limb leads or >1.5 mm in lead V1.^[7]^ An S1Q3T3 pattern was defined as the presence of an S wave in lead I and Q wave in lead III in association with T inversion in lead III.^[7]^ A low voltage was defined as the greatest overall deflection of the QRS complex of ≤5 mm in all limb leads.^[7]^ The QT interval was measured from the onset of the QRS complex to the end of the T wave in leads V1 and V6 (Fig. 1) as described previously.^[15]^ The QTc was calculated using the Bazett formula (QTc = QT/√RR). QTc difference (V1−V6) was calculated by subtracting the QTc in lead V6 from that in lead V1. These intervals should be determined as the mean value from at least 3 to 5 cardiac cycles.^[15]^ However, in the study patients with no available rhythm strips of the leads with the longest intervals, only 1 or 2 cardiac cycles were used. Two physicians (C. H. K. and S. J. P.) independently measured these intervals by using on-screen electrocardiogram analysis equipment (Cardio Calipers 3.3; Iconico.com, New York, NY). The intraobserver and interobserver variabilities of the QTc in V1 had the intraclass correlation coefficients of 0.98 [95% confidence interval (95% CI), 0.97–0.99] and 0.99 (95% CI, 0.98–0.99), respectively, and for the QTc in V6, the coefficients were 0.97 (95% CI, 0.96–0.98) and 0.98 (95% CI, 0.97–0.98), respectively. The differences between the 2 observers were resolved by consensus.

## 3 Statistical analysis

Statistical analyses were performed using SPSS 17 software (SPSS Inc., Chicago, IL). The data were expressed as the mean ± standard deviation for continuous variables and as frequencies with percentages for categorical variables. Continuous variables were compared by using a Student *t* test or Mann–Whitney test, and categorical variables using a Chi-square test, test, or Fisher exact test. Receiver operating characteristics analysis was used to determine the optimal cutoff values of continuous variables for the diagnosis of PTE. All *P* values were 2-tailed, and a *P* < .05 was considered statistically significant.

## 4 Results

Among 89 patients with suspected PTE enrolled in this study, CTA identified 45 patients with acute PTE and 44 without PTE. Among the patients in whom PTE was excluded by CTA findings, the final diagnosis was as follows: nonspecific dyspnea in patients who underwent orthopedic surgery (n = 10) or bed-ridden patients (n = 4), pneumonia (n = 7), chronic obstructive lung disease (n = 4), lung cancer (n = 5), other cancers (n = 6), and congestive heart failure (n = 8). There were no patients with coronary artery disease.

The baseline characteristics of the 2 groups are summarized in Table 1. The mean age was 67.9 ± 14.9 years, and there were 53 females (56.7%). The age, sex, and underlying medical history except hypertension did not differ between the 2 groups. The chief complaint of most patients in both groups was dyspnea or dyspnea on exertion, and did not differ significantly between the 2 groups. The d-dimer and high-sensitivity troponin I levels were significantly higher in patients with PTE than in those without (*P* < .05).

**Table 1:**
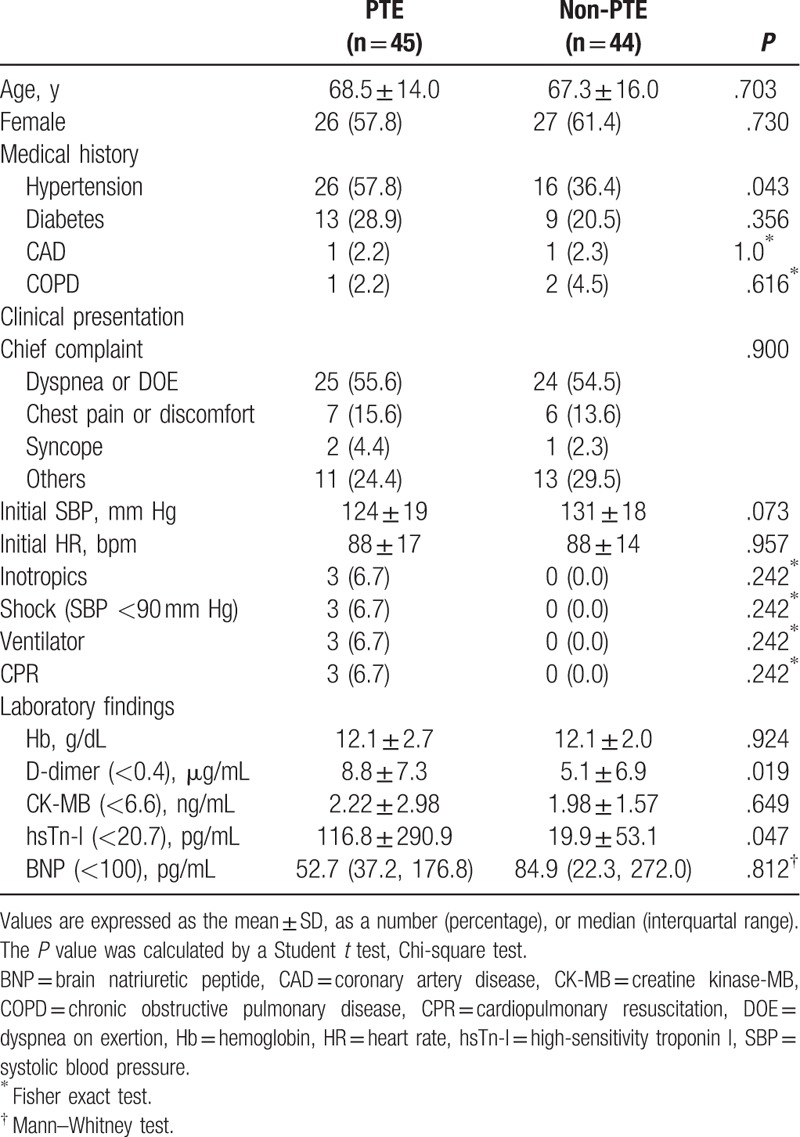
Baseline clinical characteristics of patients with or without pulmonary thromboembolism.

**Figure 2:**
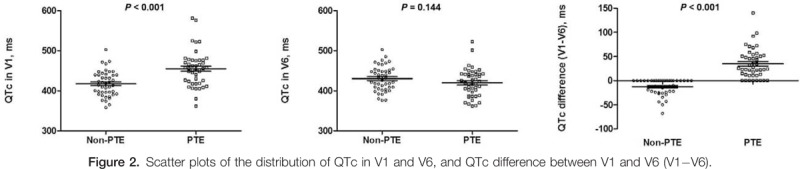
Scatter plots of the distribution of QTc in V1 and V6, and QTc difference between V1 and V6 (V1−V6).

The measured parameters and morphologic characteristics of the 2 groups are summarized in Table 2. The QT and QTc in lead V1 were significantly greater in the PTE group than in the non-PTE group. However, the QT and QTc in lead V6 did not differ significantly between the 2 groups. Therefore, the QTc difference (V1−V6) was significantly larger in the PTE group than in the non-PTE group (34.8 ± 30.5 vs −12.5 ± 16.6 ms, *P* < .001); no patients in the non-PTE group had positive values. Figure 2 shows scatter plots of the distribution of the QTc in leads V1 and V6, and the QTc difference (V1−V6). In morphological analysis, T wave inversion in lead V1 or lead III was significantly higher in the PTE group than in the non-PTE group. The prevalence of other ECG morphological parameters including S1Q3T3 did not differ significantly between the 2 groups. The prevalence of the S1Q3T3 pattern was 17.8% in the PTE group, which was not significantly different from that (11.4%) in the non-PTE group. Its sensitivity and positive predictive value for identifying PTE were 17.8% and 61.5%, respectively.

**Table 2:**
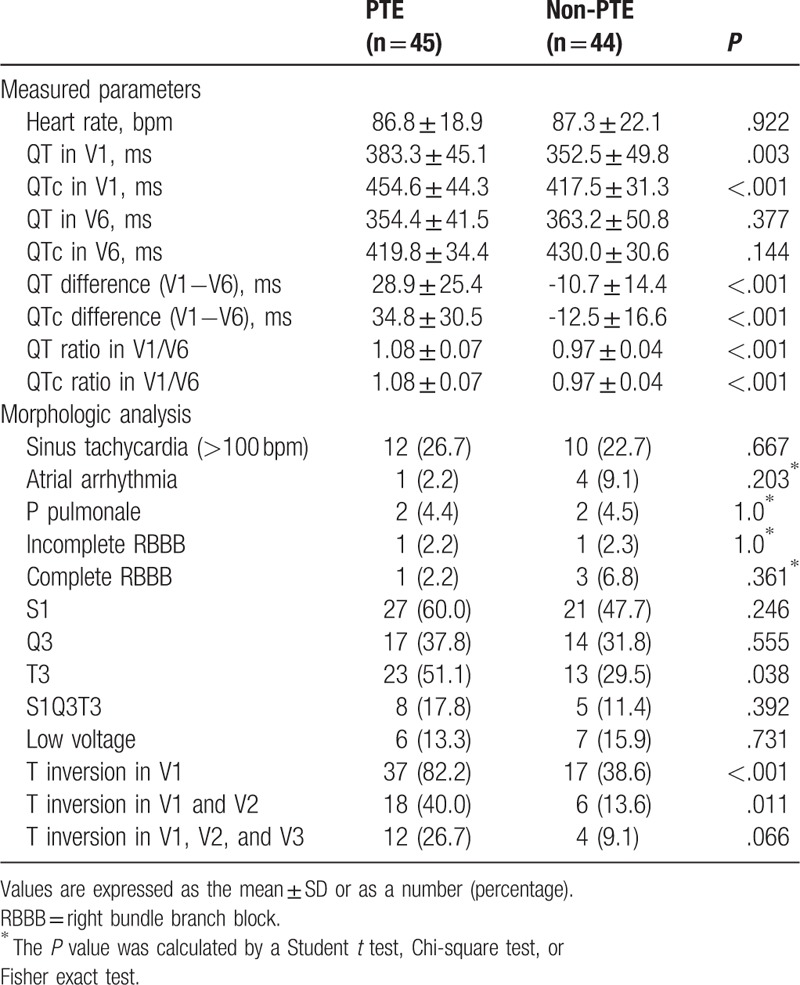
Electrocardiographic characteristics of patients with or without pulmonary thromboembolism.

**Figure 3:**
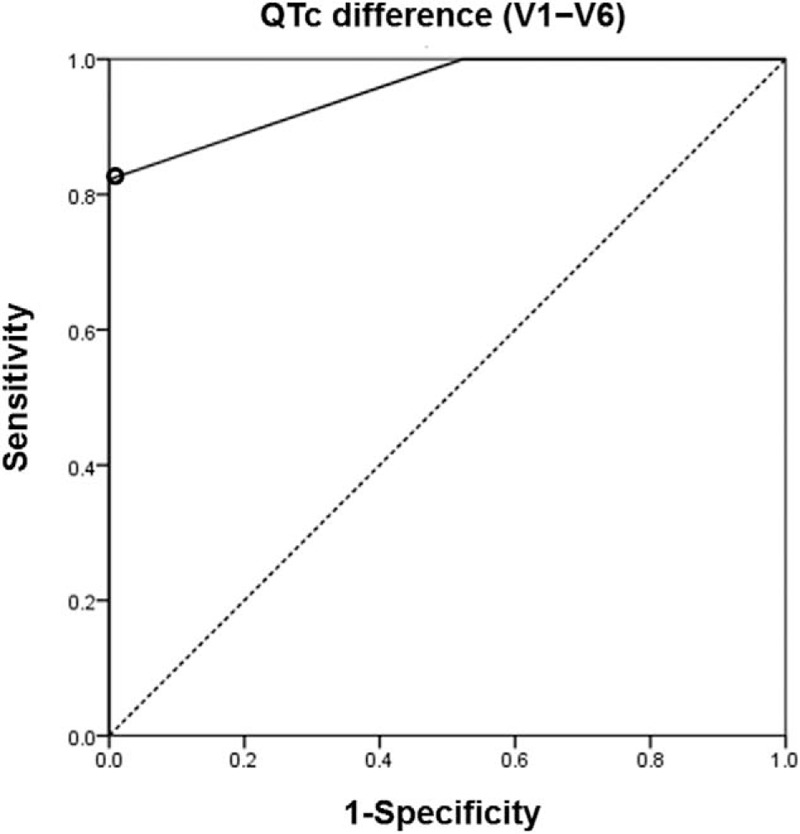
Optimal cutoff value of QTc difference between V1 and V6 (V1−V6) was determined as 20 ms with ROC analysis.

The predictive values of the ECG parameters are summarized in Table 3. For the maximal accuracy in predicting acute PTE, the biggest QTc difference (V1−V6) was 20 ms, with a sensitivity of 82.2% and specificity of 100% (area under the curve = 0.954; 95% CI, 0.914–0.993, *P* < .001) in receiver operating characteristics analysis (Fig. 3). T wave inversion in lead V1 was the most sensitive morphologic abnormality associated with PTE, with a sensitivity of 82.2% and specificity of 61.4%, and T wave inversion in lead III was the second most sensitive predictor, with a sensitivity of 51.1% and specificity of 70.5%.

**Table 3:**
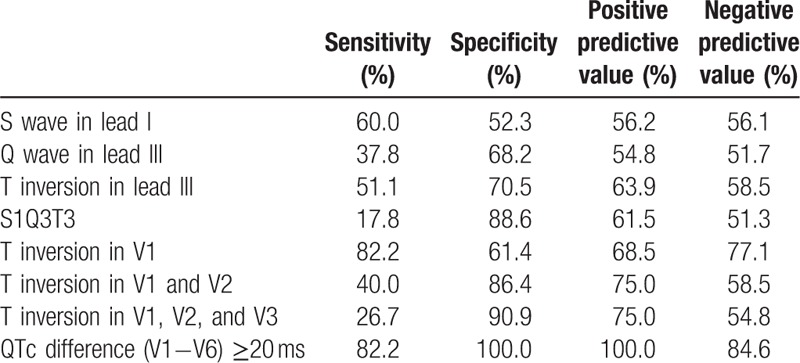
Predictive value of electrocardiographic markers for identifying pulmonary thromboembolism.

Among the 89 patients, 56 (62.9%) underwent echocardiography (35 in the PTE group and 21 in the non-PTE group) during the evaluation period. The V_max_ of tricuspid regurgitation (2.84 ± 0.81 vs 2.94 ± 0.56 m/s, *P* = .587) and RV systolic pressure (42.1 ± 24.1 vs 44.2 ± 15.9 mm Hg, *P* = .700) did not differ significantly between the 2 groups. The prevalence of RV dysfunction was higher in the PTE group than in the non-PTE group, but the difference did not reach statistical significance [13 (37.1%) in the PTE group vs 4 (14.3%) in the non-PTE group, *P* = .067].

Three patients were defined as massive PTE. QTc difference (V1−V6) (42.5 ± 28.5 ms) of massive PTE patients was bigger than that (34.2 ± 30.9 ms) of other nonmassive 42 PTE patients insignificantly (*P* > .05).

## 5 Discussion

The major findings of this study are as follows: the QTc in lead V1 and QTc difference (V1−V6) were significantly greater in PTE patients than in the non-PTE group, and QTc difference (V1−V6) of ≥20 ms was the best predictor of PTE. We expect this proposed ECG marker to be useful for differentiating patients with and without acute PTE in the acute clinical setting.

A number of ECG abnormalities are associated with acute PTE. Although the S1Q3T3 pattern is the best-known abnormality in the acute phase of PTE,^[16]^ its diagnostic value is debatable.^[7,8,17]^ In the present study, the S1Q3T3 pattern did not have any diagnostic value for acute PTE. Precordial T wave inversion has been known as a specific ECG marker of acute PTE with a positive predictive value of around 90%.^[8,11]^ However, our results were not consistent with these previous results. In this study, T wave inversion in V1 was frequent in the PTE group, but the prevalence of T inversion in leads V1–3 was low. Therefore, the positive predictive value of T inversion in leads V1–3 was 75%, but its sensitivity was very low (26.7%). Other ECG abnormalities, including sinus tachycardia, atrial arrhythmias, P pulmonale, and right bundle branch block, were not specific for acute PTE in this study.

These ECG abnormalities are associated with RV strain in the acute phase of PTE.^[18]^ This association can be explained by the observation that RV pressure overload in acute PTE affects right-sided cardiac conduction and repolarization to a greater degree than does chronically elevated RV pressure.^[18,19]^ An animal study also suggested that experimental acute pulmonary hypertension can produce RV subendocardial ischemia, which may explain the impairment of right-sided cardiac conduction and repolarizatioin.^[13]^ The mechanism of RV ischemia may be explained that dilated RV derived from acute PTE compresses the interventricular septum or the right coronary artery.^[20]^ We speculated that when RV repolarization is impaired in acute PTE, there should be a heterogeneity of repolarization between both ventricles. Then, the QT interval would be prolonged in lead V1 representing the RV side, but not in lead V6 representing the left ventricle. Therefore, in this study, we measured the QTc difference (V1−V6) in patients with suspected acute PTE and evaluated its prevalence and diagnostic value. Other studies also reported repolarization abnormality in acute PTE.^[21,22]^ Icli et al^[21]^ introduced that Tpeak-Tend interval, which is an indicator of transmural myocardial repolarization, could be a useful method in early risk stratification in patients with acute PTE. Ding et al^[22]^ reported that corrected QT dispersion is increased in acute PTE, and high corrected QT dispersion suggest poor prognosis. As inverted precordial T waves are caused by functional or structural RV changes attributable to pressure overload,^[23]^ abnormality of repolarization can also be explained by these mechanisms.

Clinical characteristics including the chief complaint did not differ significantly between PTE and non-PTE patients in this study. In addition, although the d-dimer and high-sensitivity troponin I levels were significantly higher in the PTE group than in the non-PTE group, the mean values of these biomarkers in non-PTE patients exceeded the normal range. Echocardiographic examination revealed a high prevalence of RV enlargement or dysfunction in PTE patients, but the difference between the PTE and non-PTE groups was not significant. Because it is difficult to diagnose acute PTE on the basis of clinical presentation and biomarkers, our new ECG finding, a QTc difference (V1−V6) of ≥20 ms, can help to tentatively diagnose acute PTE and decide whether to perform CTA for a definitive diagnosis.

There were some limitations in our study. First, our findings resulted from a small study population. Actually, by crude estimation, a sample size of 500 is needed to achieve the precision level of 0.05 for sensitivity. However, we could not enroll the needed sample size. So, further study should be performed in large study population. In addition, our study was performed in a Korean population at a single center. Therefore, there might be selection bias and recall bias. Thus, it needs to be validated in other populations. A chart recorder was used for all ECGs, and the QTc might be ambiguous or might depend on the observer. However, we tried to reduce the errors in QTc measurements by using on-screen electrocardiogram analysis equipment and performing repeated measurements by 2 physicians. It may be difficult to clarify the distinction between 20 ms difference in QTc difference (V1−V6) visually. However, almost all patients in the PTE group but none in the non-PTE group had positive values of QTc difference (V1−V6). Therefore, an accurate measurement of QTc difference (V1−V6) will provide an excellent diagnostic opportunity to differentiate patients with and without acute PTE.

## 6 Conclusion

QTc difference (V1−V6) is a distinguishing feature in patients with acute PTE. QTc difference (V1−V6) of ≥20 ms is a significant marker to identify acute PTE in emergency care settings.
